# Combined *TP53* Codon 72 Genotype and p53 Expression in Association with Vitamin D Benefit in Digestive Tract Cancer: A Post-Hoc Study of the AMATERASU Trial

**DOI:** 10.3390/cancers18203286

**Published:** 2026-10-12

**Authors:** Soichiro Fukuzato, Taisuke Akutsu, Hironori Ohdaira, Yutaka Suzuki, Mitsuyoshi Urashima

**Affiliations:** 1 Division of Molecular Epidemiology, Jikei University School of Medicine, Tokyo 105-8461, Japan; sunshine1.324@jikei.ac.jp (S.F.);; 2 Department of Surgery, International University of Health and Welfare Hospital,Tochigi 329-2763, Japan

**Keywords:** *TP53* codon 72 polymorphism, p53, digestive tract cancer, vitamin D

## Abstract

**Background**: Accumulating evidence suggests that vitamin D may influence cancer prognosis. We aimed to explore whether the *TP53* codon 72 polymorphism (p.Pro72Arg; rs1042522) may be associated with differential effects of vitamin D supplementation on digestive tract cancer prognosis. **Methods**: This post hoc analysis of the AMATERASU randomized controlled trial included patients in whom both germline *TP53* codon 72 genotype and p53 immunohistochemistry (IHC) assessment were available. Patients were stratified according to *TP53* codon 72 genotype and p53 IHC expression. The association between vitamin D supplementation and relapse-free survival (RFS) was evaluated using Cox proportional hazards regression models within each subgroup. **Results**: A total of 333 participants (79.9%) out of 417 in the AMATERASU trial were included in the analysis. RFS was comparable between the vitamin D and placebo groups across the *TP53* codon 72 genotype subgroups. The vitamin D group was significantly associated with more favorable RFS compared with the placebo group in patients with both the Arg/Arg genotype and high p53 IHC expression (HR, 0.32; 95% CI, 0.12–0.87; *p* = 0.03). However, with a fragility index of 2 and a non-significant three-way interaction among vitamin D supplementation, p53 IHC expression, and the Arg/Arg genotype (*p* for interaction = 0.09), these findings remain fragile despite reaching statistical significance, warranting careful interpretation. **Conclusions**: Vitamin D supplementation was associated with more favorable RFS among patients with digestive tract cancer who had both the Arg/Arg genotype and high p53 IHC expression. These exploratory findings warrant further investigation in prospective studies.

## Simple Summary

Vitamin D supplementation may reduce the risk of cancer recurrence, but the potential benefits may differ among patients with digestive tract cancer (esophagus, stomach, small bowel, and colorectal). Both patient-related genetic factors and tumor-related biological characteristics may help explain these differences. In this study, we investigated whether a patient’s germline *TP53* codon 72 genotype, together with tumor p53 protein expression, was associated with the potential benefit of vitamin D supplementation in patients with digestive tract cancer who participated in the AMATERASU randomized clinical trial. By examining these germline and tumor characteristics together, we aimed to identify patient and tumor profiles that are associated with a greater or smaller benefit from vitamin D supplementation. Our findings may provide new insights into how host genetic factors and tumor biology may jointly relate to the effectiveness of vitamin D and help generate hypotheses for future studies of personalized cancer treatment.

## 1. Introduction

Digestive tract cancers are among the most common cancers worldwide and carry a poor prognosis even after curative resection [1,2]. Despite multimodal treatment strategies, postoperative recurrence remains a significant clinical challenge. Indeed, a systematic review of randomized controlled trials comparing surgery alone with surgery plus adjuvant chemotherapy showed that the surgery plus adjuvant chemotherapy group experienced recurrence or death in 10% of patients within the first 6 months post-randomization and in an additional 14% between 6 months and 1 year [3].

Recently, an association between vitamin D and cancer prognosis has been demonstrated in some clinical investigations [4,5,6,7]. The principal molecular mechanism underlying the antitumor activity of vitamin D involves the binding of its active form, 1,25-dihydroxyvitamin D, to the vitamin D receptor (VDR), which regulates the transcription of target genes involved in the suppression of angiogenesis, induction of apoptosis, and cell cycle arrest [8,9].

Our research group has previously demonstrated that vitamin D supplementation was significantly associated with favorable prognosis in patients with digestive tract cancer exhibiting nuclear p53 staining by immunohistochemistry (IHC) [10,11]. The *TP53* tumor suppressor gene, located on chromosome 17p13.1, encodes the p53 protein, which plays a central role in the cellular response to genotoxic stress through the regulation of cell cycle arrest, apoptosis, and DNA repair [12,13]. IHC assessment of p53 protein expression is strongly associated with *TP53* missense mutations [14,15,16,17,18]. *TP53* missense mutations are known to impair the capacity of p53 protein to bind to the promoter regions of various downstream target genes. The inability of missense mutant p53 to transactivate the *MDM2* promoter—encoding the E3 ubiquitin ligase responsible for p53 protein ubiquitination and proteasomal degradation—results in the escape of mutant p53 from *MDM2*-mediated degradation and its consequent aberrant nuclear accumulation [19,20]. Several interactions between vitamin D and p53 have been demonstrated. Both p53 and active vitamin D participate in the regulation of the *MDM2* gene, since the promoter region of *MDM2* contains vitamin D response elements [21,22]. Furthermore, vitamin D-induced cell cycle arrest leads to the accumulation of cells in the G0 phase in a p53-dependent manner [23].

A well-characterized single nucleotide polymorphism at codon 72 of the *TP53* gene results in an amino acid substitution of proline to arginine (p.Pro72Arg; rs1042522). A recent study has demonstrated that missense mutations with Arg/Arg genotype are associated with a poor prognosis in patients with ovarian cancer [24]. However, the association between the *TP53* codon 72 polymorphism and vitamin D remains uninvestigated. The present study aimed to explore whether the *TP53* codon 72 polymorphism may be associated with differential effects of vitamin D supplementation on digestive tract cancer prognosis and to discuss potential biological mechanisms underlying this association based on previously reported evidence.

## 2. Materials and Methods

### 2.1. Study Design and p53 Expression Assessment

This study was conducted as a post hoc analysis of the AMATERASU trial, a randomized, double-blind, placebo-controlled trial investigating the effects of vitamin D supplementation in patients with digestive tract cancers, conducted at the International University of Health and Welfare Hospital in Japan. In the original trial, patients aged 30 to 90 years with cancers of the digestive tract (esophagus, stomach, small bowel, and colorectal), stages I to III, were recruited. The primary analysis was performed according to the intention-to-treat principle, with patients analyzed according to their original randomized treatment assignment [10]. IHC staining for p53 and its evaluation methods were performed as previously described [11]. IHC for p53 was performed with the Histofine Histostainer 36A (Nichirei Biosciences Inc., Tokyo, Japan). IHC with the primary antibody p53 DO-7 (Nichirei Biosciences Inc., Tokyo, Japan) was performed according to the manufacturer’s protocols. The degree of p53 expression based on immunoreactive nuclear cells in the cancer glandular component was evaluated by a researcher, who was blinded to randomized group and outcomes. p53 IHC expression was categorized into four groups based on the proportion of tumor cells with nuclear staining: 0%, <0% to ≤10%, <10% to ≤50%, and <50%. Cut-off points of 10% and 50% were applied based on previous studies. A 10% cut-off point has been shown to be 84–100% sensitive and 86–97% specific for detecting *TP53* missense mutations [14], and was identified as the most commonly chosen threshold in a meta-analysis of 36 studies on p53 IHC for *TP53* gene mutations [25]. Additionally, a 50% cut-off point was selected because a 50% threshold demonstrated 100% specificity for association with high-grade dysplasia in Barrett’s esophagus [26], as well as high diagnostic accuracy for *TP53* mutations in colorectal cancer, with a sensitivity of 0.85 and a specificity of 0.77 [27]. These cut-off criteria were prespecified.

### 2.2. DNA Extraction and TP53 Sequencing

Genomic DNA was extracted from tumor tissue samples by SRL Inc. (Tokyo, Japan) and subsequently stored at −80 °C until use for somatic *TP53* sequencing. Before starting supplements, peripheral blood was drawn and sent to SRL Inc. (Tokyo, Japan) to purify genomic DNA as previously described [10]. To assess concordance between germline and tumor-derived *TP53* codon 72 genotypes, direct sequencing of tumor-derived genomic DNA was performed in a subset of patients for whom tumor tissue samples were available. Polymerase chain reaction (PCR) amplification was performed using primers as previously described [28]. PCR was carried out using PrimeSTAR^®^ Max DNA Polymerase Ver.2 (Takara Bio Inc., Kusatsu, Japan) under the following thermocycling conditions: initial denaturation followed by 30 cycles of denaturation at 98 °C for 10 s, annealing at 55 °C for 5 s, and extension at 68 °C for 5 s. PCR products were electrophoresed on a 1% agarose gel. Sanger sequencing was performed by FASMAC Co., Ltd. (Atsugi, Japan). The obtained sequences were aligned to the human reference genome (GRCh38) and analyzed for the *TP53* codon 72 polymorphism and somatic mutations. Patients were categorized into the RR vs. PR/PP genotype groups based on previous findings demonstrating that the RR genotype was associated with significantly worse prognosis in ovarian cancer patients harboring *TP53* missense mutations [24]. Biologically, p53 functions as a tetramer; therefore, to exclude potential dominant-negative effects of the Pro allele [12,13], we specifically compared patients carrying homozygous Arg alleles (RR) with those carrying at least one Pro allele (PR/PP).

### 2.3. Statistical Analysis

Differences in baseline characteristics between genotype groups were assessed using the Mann–Whitney U test for continuous variables and Fisher’s exact test for categorical variables. Deviation of genotype frequencies from Hardy–Weinberg equilibrium was evaluated using the exact test. Relapse-free survival (RFS) was used for survival analysis, and its evaluation period was determined as previously described [10]. The 5-year RFS was estimated using the Kaplan–Meier method. RFS was estimated using the Nelson–Aalen cumulative hazard estimator, and survival curves were compared accordingly. Hazard ratios (HRs) and their 95% confidence intervals (CIs) were calculated using the Cox proportional hazards regression models. To formally assess potential effect modification, three-way interaction terms for vitamin D supplementation, *TP53* codon 72 genotype, and p53 IHC expression status were included in Cox proportional hazards regression models. To assess the statistical stability of the observed difference in RFS within the key subgroup (RR genotype with p53 IHC expression <50% stratum), we performed a fragility analysis for time-to-event data. Beginning with the censored patient with the shortest follow-up time in the vitamin D group, individual outcomes were sequentially reclassified as events. A log-rank test was re-analyzed at each step. The fragility index was defined as the minimum number of outcome reclassifications required to alter the statistical significance from *p* < 0.05 to *p* > 0.05. The proportional hazards assumption for the Cox regression models was evaluated using Schoenfeld residuals. All statistical analyses were performed using Stata/SE version 19.0 (StataCorp LLC, College Station, TX, USA). All statistical tests were two-sided, and a *p*-value of less than 0.05 was considered statistically significant.

### 2.4. Ethical Considerations

This study was approved by the Institutional Review Board of The Jikei University School of Medicine (approval number: 36-192[12301]) and the International University of Health and Welfare Hospital (approval number: 13-B-263). An opt-out approach was implemented by publicly disclosing study information on the institutional website, allowing patients the opportunity to decline participation. The study adhered to the principles of the Declaration of Helsinki (as revised in 2013).

## 3. Results

### 3.1. Patient Selection and Study Population

**Figure 1:**
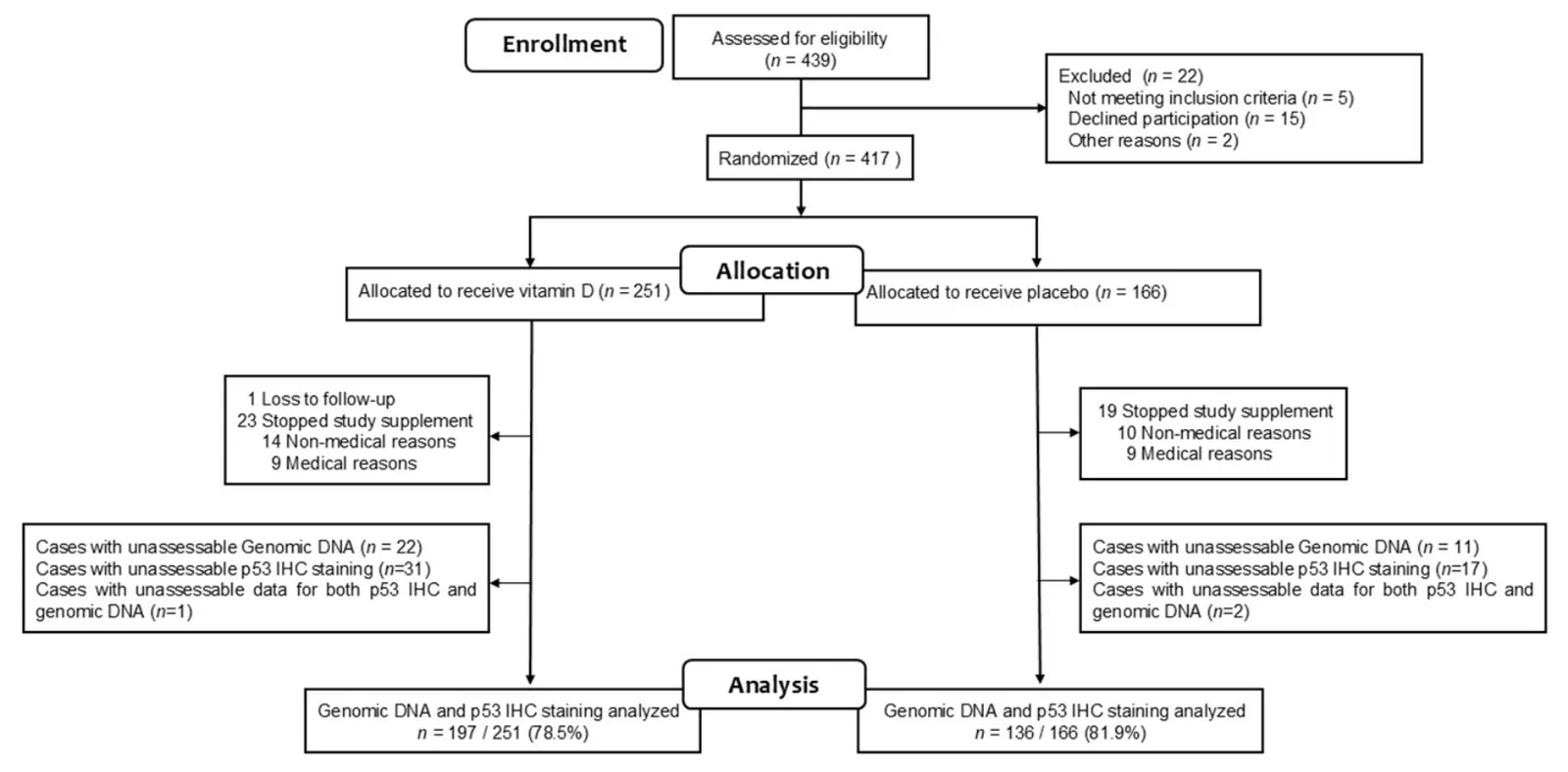
Flowdiagram.

A total of 333 patients were included in this post hoc analysis, representing 79.9% of the 417 participants enrolled in the AMATERASU randomized controlled trial. 84 patients were excluded due to unassessable genomic DNA sequencing (*n* = 33), unassessable p53 IHC staining (*n* = 48), or both (*n* = 3). Of the 333 included patients, 197 had been assigned to the vitamin D group and 136 to the placebo group in the original trial (Figure 1). The characteristics of patients who were included in and excluded from the present study are presented in Table S1.

### 3.2. TP53 Codon 72 Genotype Distribution and Baseline Characteristics According to TP53 Codon 72 Genotype

Among the 333 patients included in the analysis, 142 (42.6%) were homozygous for the Arg72 allele (RR genotype), 145 (43.5%) were heterozygous (PR genotype), and 46 (13.8%) were homozygous for the Pro72 allele (PP genotype). The genotype distribution did not deviate significantly from Hardy–Weinberg equilibrium (*p* = 0.40). The patients were divided into two genotype groups: 142 (42.6%) with the RR genotype and 191 (57.4%) with the PR/PP genotype (Table 1 and Table S2). There were no statistically significant differences in baseline characteristics between the two genotype groups (RR vs. PR/PP) (Table 1).

### 3.3. RFS According to TP53 Codon 72 Genotype in the Placebo Group

**Figure 2:**
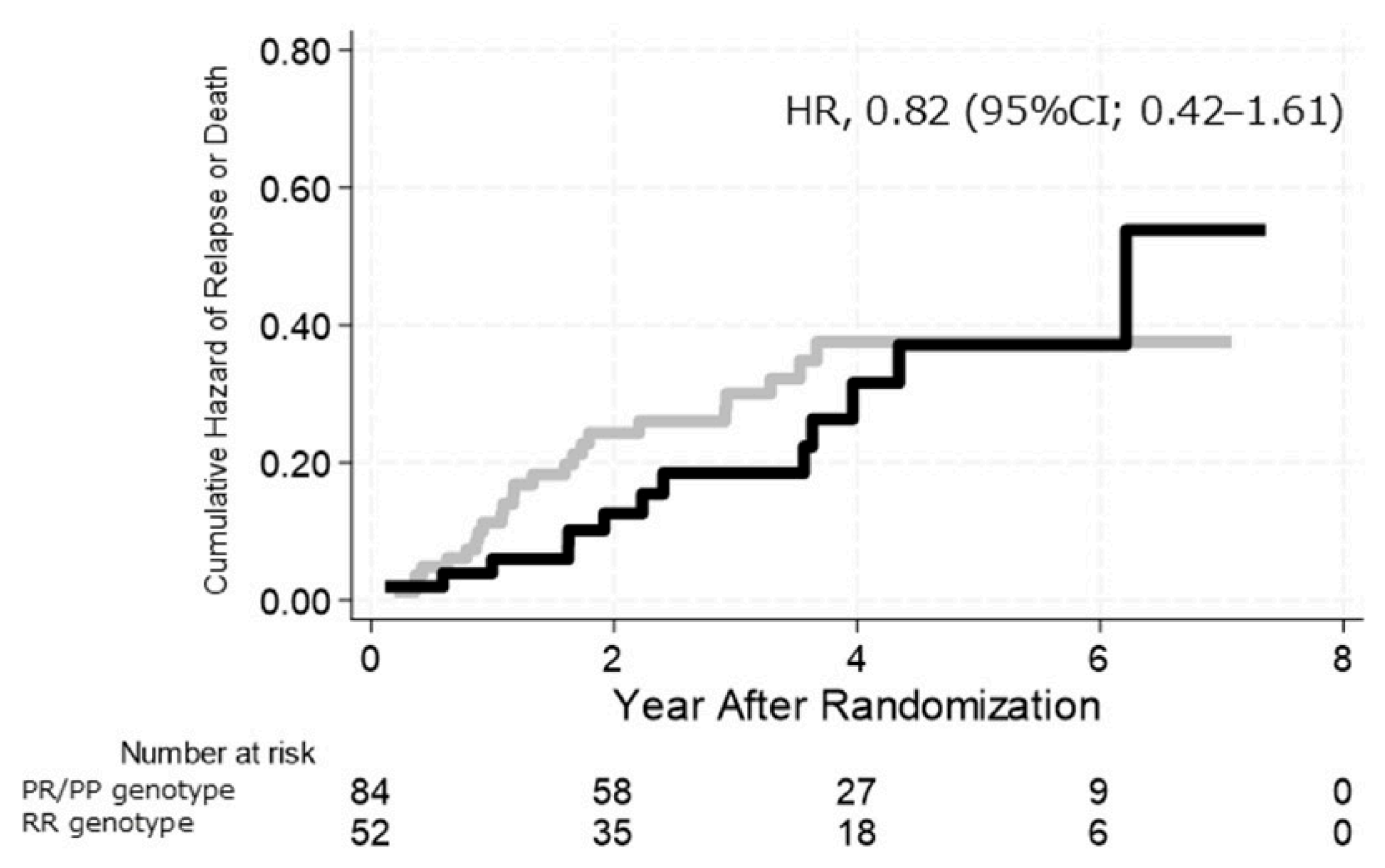
Subgroup of placebo compare RR with PR/PP genotype. Nelson–Aalen cumulative hazard curves for RFS in the placebo group, stratified by *TP53* codon 72 genotype. The black line represents patients with the RR genotype, and the gray line represents patients with the PR/PP genotype. HRs and 95% CIs are presented for the RR genotype group with the PR/PP genotype as the reference. RFS, relapse-free survival; HR, hazard ratio; CI, confidence interval.

To assess the prognostic association of the *TP53* codon 72 genotype independent of vitamin D supplementation, RFS was compared between the RR genotype group and the PR/PP genotype group within the placebo arm. There was no significant difference in RFS between the RR genotype and PR/PP genotype groups (HR, 0.82; 95% CI, 0.42–1.61). The 5-year RFS rates were 68.5% and 68.5%, respectively (Figure 2).

### 3.4. Effect of Vitamin D Supplementation on RFS According to TP53 Codon 72 Genotype

**Figure 3:**
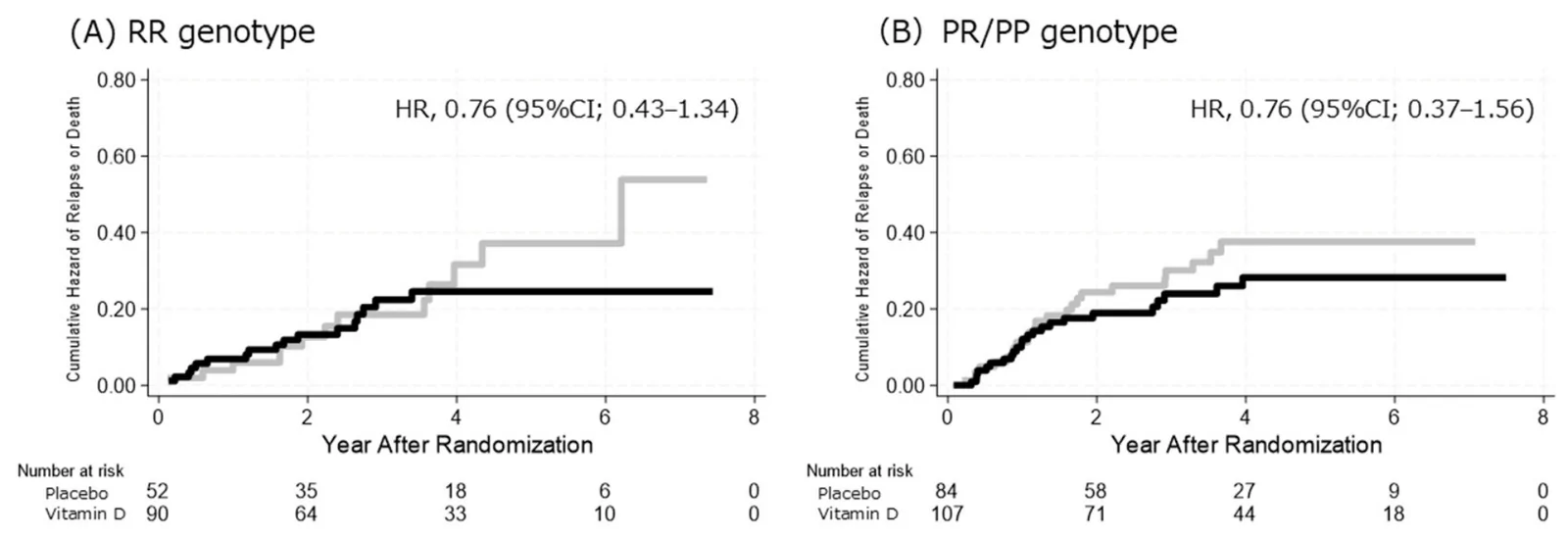
Subgroup of RR and PR/PP genotype. (**A**) Nelson–Aalen cumulative hazard curves for RFS in the subgroup of patients with the RR genotype. (**B**) Nelson–Aalen cumulative hazard curves for RFS in the subgroup of patients with the PR/PP genotype. In both panels, the black line represents the vitamin D supplementation group, and the gray line represents the placebo group. The HRs and 95% CIs of the vitamin D group compared with the placebo group are shown in each panel. RFS, relapse-free survival; HR, hazard ratio; CI, confidence interval.

The association between vitamin D supplementation and RFS was subsequently examined within each genotype subgroup. There was no significant difference in RFS between the vitamin D and placebo groups in either the RR genotype group (HR, 0.76; 95% CI, 0.43–1.34) or the PR/PP genotype group (HR, 0.76; 95% CI, 0.37–1.56). The 5-year RFS rates were 78.1% and 68.5%, respectively, in the RR genotype group, and 75.3% and 68.5%, respectively, in the PR/PP genotype group (Figure 3).

### 3.5. Subgroup Analysis Stratified by p53 IHC Expression and TP53 Codon 72 Genotype Subgroups

**Figure 4:**
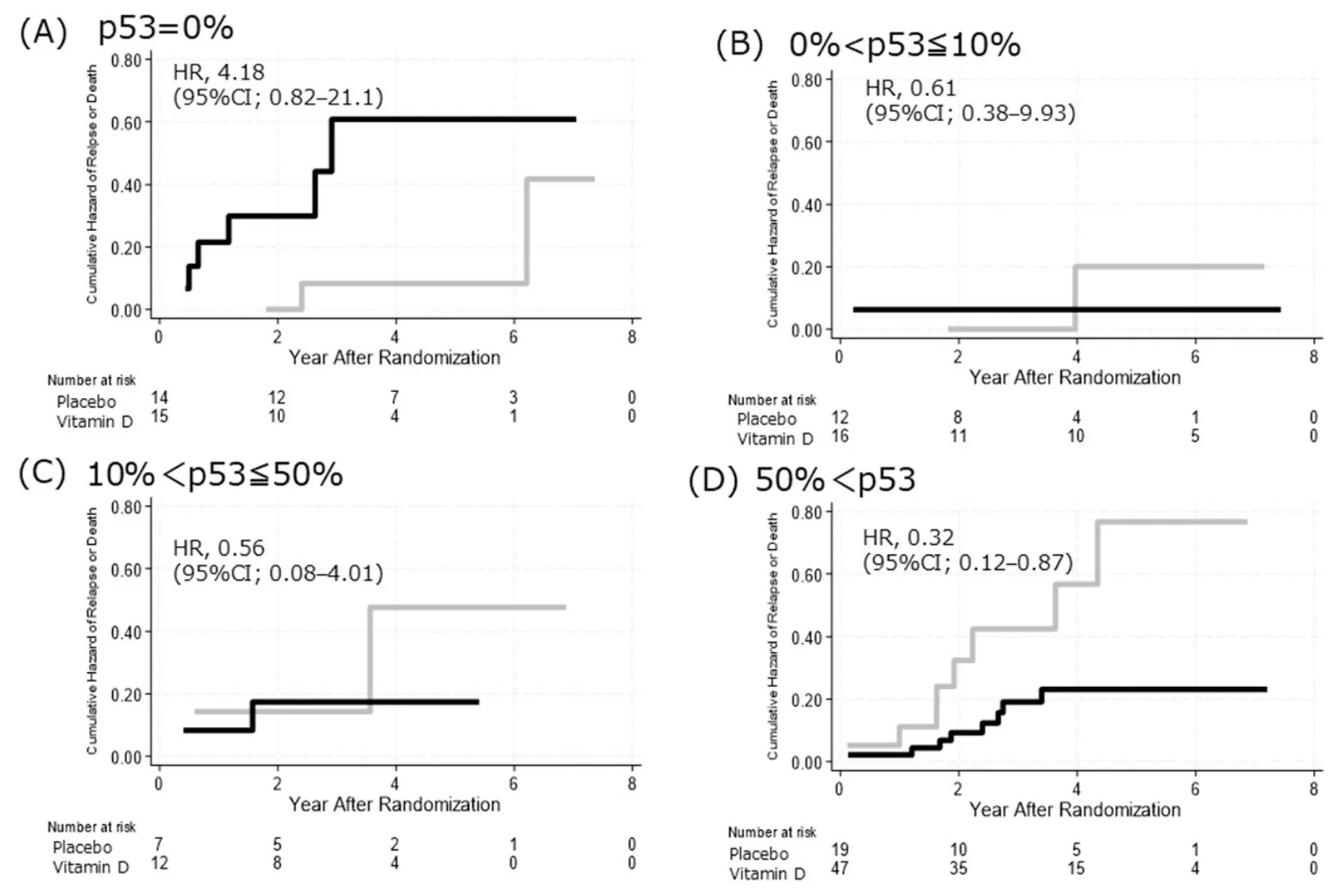
Subgroup of RR genotype and p53 IHC status. (**A**–**D**) Nelson–Aalen cumulative hazard curves for RFS in patients with the RR genotype, according to p53 expression: (**A**) 0%, (**B**) <0 to ≤10%, (**C**) <10 to ≤50%, and (**D**) <50%. Black line: vitamin D supplementation group; gray line: placebo group. The HRs and 95% CIs of the vitamin D group compared with the placebo group are shown in each panel. RFS, relapse-free survival; HR, hazard ratio; CI, confidence interval.

To further investigate the association between vitamin D supplementation and RFS, subgroup analyses were performed based on the combination of *TP53* codon 72 genotype and p53 IHC expression. The proportional hazards assumption was met for all hazard curves based on Schoenfeld residuals. A statistically significant association between vitamin D supplementation and favorable RFS was observed only in the RR genotype group with p53 IHC expression <50% stratum (HR, 0.32; 95% CI, 0.12–0.87; *p* = 0.03). The 5-year RFS rates in the vitamin D and placebo groups across the four p53 IHC expression strata were 52.4% and 91.7%, respectively, in the 0% stratum; 93.8% and 80.0%, respectively, in the <0% to ≤10% stratum; 83.3% and not reached, respectively, in the <10% to ≤50% stratum; and 79.1% and 44.1%, respectively, in the <50% stratum (Figure 4). The number of events for the vitamin D vs. placebo groups across the four p53 IHC expression strata were as follows: 6/15 (40.0%) vs. 2/14 (14.3%) in the 0% stratum; 1/16 (6.3%) vs. 1/12 (8.3%) in the <0% to ≤10% stratum; 2/12 (16.7%) vs. 2/7 (28.6%) in the <10% to ≤50% stratum; and 8/47 (17.0%) vs. 8/19 (42.1%) in the <50% stratum. The fragility analysis revealed a fragility index of 2 for the subgroup with the RR genotype and p53 IHC expression <50% stratum, where a statistically significant result was observed. Baseline characteristics for this subgroup are presented in Table S3. Additionally, Table S4 summarizes the HRs for the vitamin D group compared with the placebo group within this subgroup, individually adjusted for clinical stage, baseline 25-hydroxyvitamin D levels, and cancer site. Furthermore, the results of subgroup analyses stratified by cancer sites are summarized in Table S5.

**Figure 5:**
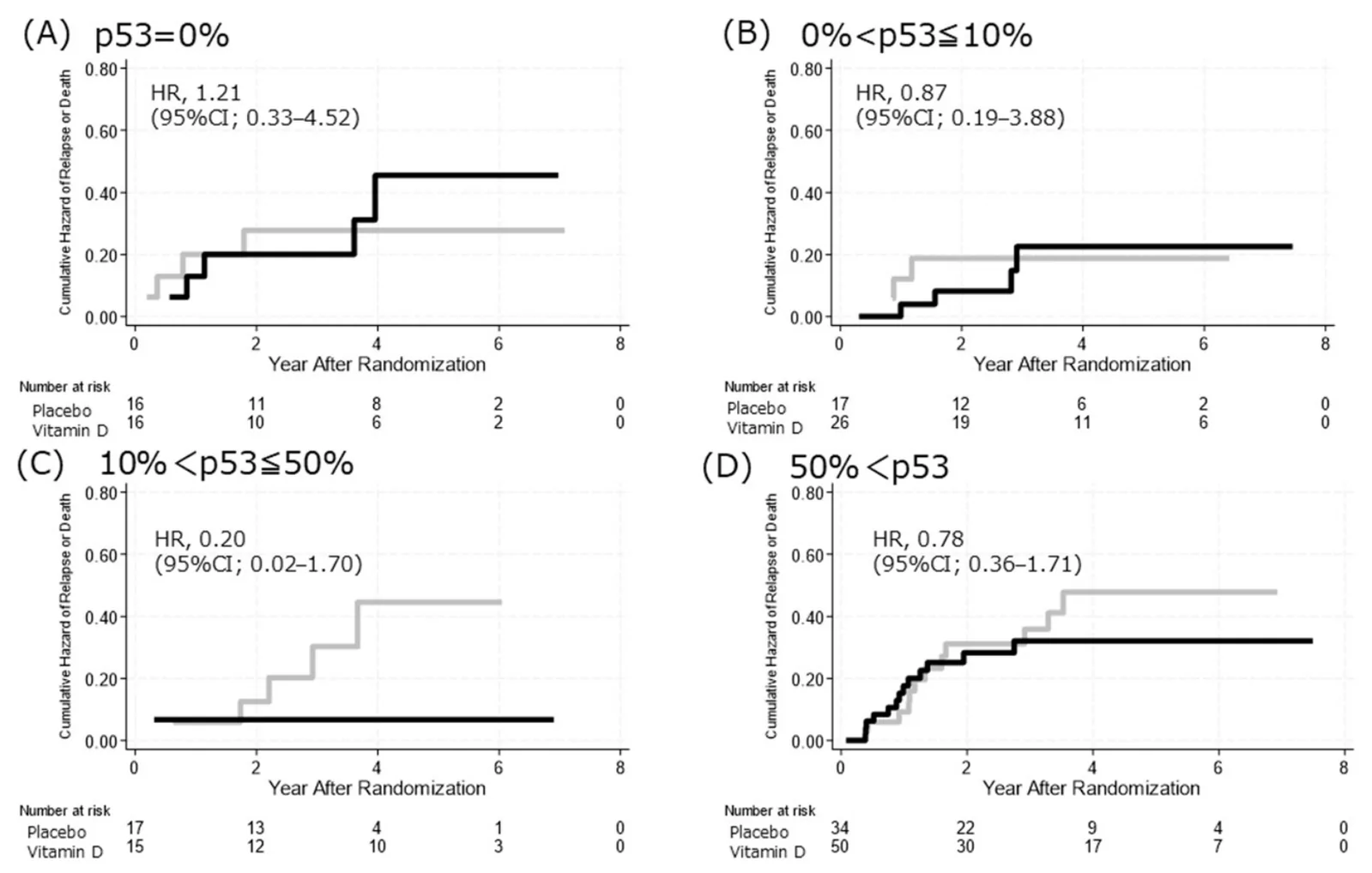
Subgroup of PR/PP genotypes and p53 IHC status. (**A**–**D**) Nelson–Aalen cumulative hazard curves for RFS in patients with the PR/PP genotype, according to p53 IHC expression: (**A**) 0%, (**B**) <0 to ≤10%, (**C**) <10 to ≤50%, and (**D**) <50%. Black line: vitamin D supplementation group; gray line: placebo group. The HRs and 95% CIs of the vitamin D group compared with the placebo group are shown in each panel. RFS, relapse-free survival; HR, hazard ratio; CI, confidence interval.

In the PR/PP genotype group, no statistically significant associations were observed across any of the p53 IHC expression strata. The 5-year RFS rates in the vitamin D and placebo groups were 61.9% and 75.0%, respectively, in the 0% stratum; 79.3% and 82.4%, respectively, in the <0% to ≤10% stratum; 93.3% and 62.6%, respectively, in the <10% to ≤50% stratum; and 72.2% and 61.4%, respectively, in the <50% stratum (Figure 5). The event counts for the vitamin D vs. placebo groups were as follows: 5/16 (31.3%) vs. 4/16 (25.0%) in the 0% stratum; 4/26 (15.4%) vs. 3/17 (17.6%) in the <0% to ≤10% stratum; 1/15 (6.7%) vs. 5/17 (29.4%) in the <10% to ≤50% stratum; and 13/50 (26.0%) vs. 12/34 (35.3%) in the <50% stratum (Figure 5). The analytical results categorized by *TP53* codon 72 genotypes (RR, PR, and PP) combined with p53 IHC expression strata are summarized in Table S6. As a sensitivity analysis, p53 IHC expression was dichotomized into two groups: ≤10% and <10%. When stratified by p53 IHC expression (≤10% vs. <10%) and *TP53* codon 72 genotype (RR vs. PR/PP), the 5-year RFS and HR for the vitamin D group vs. the placebo group were as follows: in patients with the RR genotype, 74.6% vs. 87.1%; HR, 2.14; 95% CI, 0.55–8.30 in the ≤10% stratum, and 79.9% vs. 48.4%; HR, 0.38; 95% CI, 0.18–0.91 (*p* = 0.03) in the <10% stratum (Figure S1); whereas in those with the PR/PP genotype, 72.1% vs. 78.7%; HR, 0.98; 95% CI, 0.37–2.65 in the ≤10% stratum, and 77.3% vs. 66.2%; HR, 0.67; 95% CI, 0.33–1.36 in the <10% stratum. The three-way interaction among vitamin D supplementation, p53 IHC expression (<50%), and the RR genotype was not statistically significant (*p* for interaction = 0.09). Similarly, the three-way interaction using the <10% p53 IHC threshold was not statistically significant (*p* for interaction = 0.19).

### 3.6. Comparison of TP53 Codon 72 Genotype Between Germline and Tumor Somatic DNA

Among the 121 patients in whom both germline and tumor somatic *TP53* codon 72 genotyping was successfully performed, discordance between germline and somatic *TP53* codon 72 genotypes was observed in 18 of the 121 patients (14.9%) (Table S7). High p53 IHC expression <50% showed a positive predictive value of 44.1% (26/59) and a negative predictive value of 90.0% (54/60) for identifying *TP53* missense mutations (Table S8).

## 4. Discussion

The principal finding of the present study was that vitamin D supplementation was associated with more favorable RFS in patients with the RR genotype and high p53 IHC expression. Statistically significant differences were observed in both the <50% and <10% p53 IHC expression strata. To our knowledge, this is the first study to suggest that the combination of *TP53* codon 72 genotype and p53 IHC expression may be associated with a more favorable RFS among patients receiving postoperative vitamin D supplementation for digestive tract cancers. Specifically, the interaction terms were not statistically significant; because this does not demonstrate that the combination of *TP53* codon 72 genotype and p53 IHC expression modify the therapeutic effect of vitamin D supplementation, caution should be exercised when interpreting these results. Furthermore, as this was an exploratory study, no corrections for multiple testing were performed, raising the possibility of false positives. Given this and the small sample size in these subgroups, these findings remain fragile despite reaching statistical significance, warranting careful interpretation.

There were no statistically significant differences in baseline characteristics between the two genotypes (RR vs. PR/PP). The distribution of the *TP53* codon 72 genotype observed in the present study was consistent with previously reported allele frequencies in East Asian populations [29,30]. When the prognostic association of the *TP53* codon 72 polymorphism was evaluated independently of vitamin D supplementation, RFS was comparable among the *TP53* codon 72 genotypes, consistent with prior studies [31]. Furthermore, when the association between vitamin D supplementation and RFS was assessed according to the *TP53* codon 72 genotype alone, neither the RR nor the PR/PP genotype subgroup demonstrated a statistically significant association, with numerically similar HRs observed between the vitamin D supplementation group and the placebo group. The mechanistic basis for the observed association between vitamin D supplementation, the RR genotype and high p53 IHC expression remains highly speculative and conjectural, as our current data cannot directly validate any underlying biological pathways. IHC assessment of p53 protein expression has been validated as a highly sensitive and specific surrogate marker for *TP53* missense mutations [14,15,16,17,18]. However, in the present study, high p53 IHC expression yielded a low positive predictive value of 44.1%, despite a high negative predictive value of 90.0%. This suggests that high p53 IHC expression may not be a sufficient surrogate marker for *TP53* missense mutations in the present study. Emerging evidence suggests that *TP53* missense mutations with the RR genotype result in a more profound impairment of downstream target gene transactivation, including *MDM2* and other tumor suppressor target genes, and worse prognosis compared with equivalent mutations with the PP genotype [24], attributable to a reduced capacity of missense mutant p53 with the RR genotype to bind to downstream tumor suppressor target gene promoters. Indeed, in the present study, patients with both high p53 IHC expression and the RR genotype exhibited a trend toward poorer prognosis compared with the other subgroups in the placebo group. Previous studies have reported a functional link between p53 and vitamin D [23,32,33]. In gastric cancer cells, mutant p53 has been shown to bind directly to nuclear VDR and enhance its stability, thereby potentiating 1,25(OH)_2_D-induced p21 upregulation and CDK2 suppression leading to cell cycle arrest, suggesting that the antitumor effect of vitamin D may be augmented through VDR interaction specifically in mutant p53-harboring gastrointestinal cancer cells [34]. As a potential mechanism underlying our findings, we hypothesize that vitamin D may be associated with a more favorable prognosis by modulating transcriptional activity in cancer patients harboring *TP53* missense mutations with the RR genotype. Future prospective, prespecified interventional studies should assess *TP53* codon 72 genotype and p53 expression in tumor tissue to determine whether biomarker-guided postoperative vitamin D supplementation can reduce cancer recurrence. Such studies could ultimately inform a personalized approach to postoperative vitamin D supplementation for patients who are most likely to benefit.

## 5. Limitations

Several limitations of the present study should be acknowledged. First, the present study did not demonstrate a statistically significant three-way interaction among vitamin D supplementation, *TP53* codon 72 genotype, and p53 IHC expression. Second, although IHC assessment of p53 protein expression was employed as a validated surrogate marker for *TP53* missense mutations, tumor sequencing was performed only in a subset of patients in the present study. Third, as the present study was conducted as a post hoc subgroup analysis of the AMATERASU trial, the available sample size was substantially reduced compared with the original trial. Fourth, no adjustment for multiple testing was performed; therefore, the possibility of type I errors cannot be excluded, and the findings should be considered exploratory rather than confirmatory. Fifth, all patients were recruited from a single Japanese institution, and the present findings may not be directly generalizable to other ethnic populations. Sixth, Due to the low positive predictive value of high p53 IHC expression, high p53 expression may not be a sufficient surrogate marker for missense mutations.

## 6. Conclusions

In the present study, vitamin D supplementation was associated with more favorable postoperative outcomes in the subgroup of patients with digestive tract cancers exhibiting high p53 IHC expression and the RR genotype. However, given the small sample size in this subgroup, these findings should be interpreted with caution. Furthermore, this study does not establish causal relationships and remains hypothesis-generating; therefore, prospective, adequately powered studies are required before drawing any definitive clinical conclusions.

## Figures and Tables

**Table 1:** Patient Characteristics Stratified by *TP53* Codon 72 Genotype.

	RR	PR/PP
*n* (%)	142 (42.6)	191 (57.4)
Supplement, *n* (%)		
Vitamin D	90 (63.4)	107 (56.0)
Placebo	52 (36.6)	84 (44.0)
Age, years, median (IQR)	67 (62–74)	66 (59–73)
Sex, *n* (%)		
Male	92 (64.8)	126 (66.0)
Female	50 (35.2)	65 (34.0)
BMI, kg/m^2^, median (IQR)	21.9 (20.0–23.8)	21.8 (19.8–23.9)
Double cancer, *n* (%)	7 (4.9)	6 (3.1)
Pre25OHD, ng/mL, median (IQR)	22.0 (16–28)	21.0 (16–27)
25OHD at 1 Year, ng/mL, median (IQR)	35 (19–51)	30 (20–44)
Cancer sites, *n* (%)		
Esophagus	17 (12.0)	12 (6.3)
Stomach	63 (44.4)	84 (44.0)
Small bowel	1 (0.7)	1 (0.5)
Colorectal	61 (43.0)	94 (49.2)
Clinical stage, *n* (%)		
I	65 (45.8)	78 (40.8)
II	41 (28.9)	45 (23.6)
III	36 (25.4)	68 (35.6)
Pathology, *n* (%)		
Adenocarcinoma	125 (88.0)	178 (93.2)
SCC	17 (12.0)	12 (6.3)
Others	0 (0.0)	1 (0.5)
p53, *n* (%)		
0%	29 (20.4)	32 (16.8)
>0% to ≤10%	28 (19.7)	43 (22.5)
>10% to ≤50%	19 (13.4)	32 (16.8)
>50%	66 (46.5)	84 (44.0)
Adherence, *n* (%)	125 (88.0)	171 (89.5)

Continuous variables are presented as median (interquartile range) and categorical variables are presented as number (percentage). Abbreviations: BMI, body mass index; SCC, squamous cell carcinoma; IHC, immunohistochemistry; pre25OHD, pre-study serum 25-hydroxyvitamin D level; 25OHD at 1 Year, serum 25-hydroxyvitamin D level at 1 year after study initiation.

## Data Availability

The raw data supporting the conclusions of this article will be made available by the authors on request.

## Supplementary Materials

The following supporting information can be downloaded at https://www.mdpi.com/article/10.3390/cancers18203286/s1: Table S1: Characteristics of Included and Excluded Patients in Present Study, Table S2: Patient Characteristics according to *TP53* Codon 72 Genotype, Table S3: Baseline Characteristics of Patients in the RR Genotype with p53 IHC Expression <50% stratum, Table S4: Hazard ratios for relapse-free survival in the subgroup of RR genotype with p53 IHC expression <50% stratum, adjusted individually for clinical covariates, Table S5: Hazard ratios for relapse-free survival for vitamin D supplementation versus placebo in the subgroup of RR genotype with p53 IHC expression <50% stratum, stratified by cancer site, Table S6: Subgroup analysis of relapse-free survival for vitamin D supplementation versus placebo, stratified by *TP53* codon 72 genotypes (RR, PR, and PP) and p53 IHC expression stratum, Table S7: Somatic and Germline Genome of *TP53* Codon 72 Genotypes, Table S8: p53 IHC Staining and *TP53* Somatic Mutations, Figure S1: Sensitivity Analysis Subgroup of *TP53* Codon 72 Genotype and p53 IHC Status.

## Abbreviations

The following abbreviations are used in this manuscript:

IHC	immunohistochemistry
RFS	relapse-free survival
PCR	Polymerase chain reaction
HR	hazard ratio
CI	confidence interval
VDR	vitamin D receptor
