# Supplementary material for: Combined TP53 Codon 72 Genotype and p53 Expression in Association with Vitamin D Benefit in Digestive Tract Cancer: A Post-Hoc Study of the AMATERASU Trial
Source: Cancers (Basel). 2026 Oct 12;18(20):3286. doi: 10.3390/cancers18203286 (PMC13702322; doi:10.3390/cancers18203286)
Supplement: Supplementary file 1 [file cancers-18-03286-s001.zip › cancers-4589531-supplementary.pdf]

Supplementary file

Combined TP53 Codon 72 Genotype and p53 Expression in Association with Vitamin D Benefit in Digestive Tract Cancer: A Post-Hoc Study of the AMATERASU Trial

Soichiro Fukuzato[1]; Taisuke Akutsu[1]; Hironori Ohdaira[2]; Yutaka Suzuki[2]; and Mitsuyoshi Urashima[1,*]

1 Division of Molecular Epidemiology, Jikei University School of Medicine, Tokyo 105-8461, Japan; e-mail@e-mail.com

2 Department of Surgery, International University of Health and Welfare Hospital, 537-3 Iguchi, Nasushiobara, Tochigi 329-2763, Japan; e-mail@e-mail.com

* Correspondence: urashima@jikei.ac.jp; Tel.: +81-3-3433-1111

Table S1: Characteristics of Included and Excluded Patients in Present Study – page2

Table S2: Patient Characteristics according to*TP53* Codon 72 Genotype – page 3-4

Table S3: Baseline Characteristics of Patients in the RR Genotype with p53 IHC Expression 50%< stratum – page5

Table S4: Hazard ratios for relapse-free survival in the subgroup of RR genotype with p53 IHC expression 50%< stratum, adjusted individually for clinical covariates – page6

Table S5: Hazard ratios for relapse-free survival for vitamin D supplementation versus placebo in the subgroup of RR genotype with p53 IHC expression 50%< stratum, stratified by cancer site – page6

Table S6: Subgroup analysis of relapse-free survival for vitamin D supplementation versus placebo, stratified by *TP53* codon 72 genotypes (RR, PR, and PP) and p53 IHC expression stratum. – page7

Table S7: Somatic and Germline Genome of *TP53* Codon 72 Genotypes – page 8

Table S8: p53 IHC Staining and *TP53* Somatic Mutations – page 8

Figure S1: Sensitivity Analysis Subgroup of *TP53* Codon 72 Genotype and p53 IHC Status - page 9

Table S1: Characteristics of Included and Excluded Patients in Present Study

	Included	Excluded
N (%)	333 (79.9)	84 (20.1)
Supplement, n (%)		
Vitamin D	197 (59.2)	54 (64.3)
Placebo	136 (40.8)	30 (35.7)
Age, years, median (IQR)	66 (60-74)	65 (59-74.5)
Sex, n (%)		
Male	218 (65.5)	58 (69.0)
Female	115 (34.5)	26 (31.0)
BMI, kg/m^2, median (IQR)	21.9 (19.8-23.8)	21.8 (19.8-24.0)
Double cancer, n (%)	13 (3.9)	12 (14.3)
Pre25OHD, ng/mL, median (IQR)	21.0 (16-27)	20.0(16-26)
25OHD at 1 Year, ng/mL, median (IQR)	32 (19-47)	33 (24-42)
Cancer sites, n (%)		
Esophagus	29 (8.7)	11 (13.1)
Stomach	147 (44.1)	27 (32.1)
Small bowel	2 (0.6)	0 (0.0)
Colorectal	155 (46.5)	46 (54.8)
Clinical stage, n (%)		
I	143 (42.9)	39 (46.4)
II	86 (25.8)	25 (29.8)
III	104 (31.2)	20 (23.8)
Pathology, n (%)		
Adenocarcinoma	303 (91.0)	70 (83.3)
SCC	29 (8.7)	9 (10.7)
Others	1 (0.3)	5 (6.0)
Adherence, n (%)	296 (88.9)	79 (94.0)

Continuous variables are presented as median (interquartile range) and categorical variables are presented as number (percentage). Abbreviations: BMI, body mass index; SCC, squamous cell carcinoma; pre25OHD, pre-study serum 25-hydroxyvitamin D level.

Table S2: Patient Characteristics according to *TP53* Codon 72 Genotype

	RR	PR	PP
N (%)	142 (42.6)	145 (43.5)	46 (13.8)
Supplement, n (%)			
Vitamin D	90 (63.4)	81 (55.9)	26 (56.5)
Placebo	52 (36.6)	64 (44.1)	20 (43.5)
Age, years, median (IQR)	67(62-74)	67(61-74)	63(56-69)
Sex, n (%)			
Male	92 (64.8)	93 (64.1)	33 (71.7)
Female	50 (35.2)	52 (35.9)	13 (28.3)
BMI, kg/m^2, median (IQR)	21.9(20.0-23.8)	21.7(19.7-23.7)	22.0(21.0-24.2)
Double cancer, n (%)	7 (4.9)	5 (3.4)	1 (2.2)
Pre25OHD, ng/mL, median (IQR)	22.0(16-28)	21.0(15-27)	22.0(18-27)
25OHD at 1 Year, ng/mL, median (IQR)	35 (19-51)	32 (19.5-46.5)	26.5 (20-38)
Cancer sites, n (%)			
Esophagus	17 (12.0)	11 (7.6)	1 (2.2)
Stomach	63 (44.4)	55 (37.9)	29 (63.0)
Small bowel	1 (0.7)	0 (0.0)	1 (2.2)
Colorectal	61 (43.0)	79 (54.5)	15 (32.6)
Clinical stage, n (%)			
I	65 (45.8)	53 (36.6)	25 (54.3)
II	41 (28.9)	35 (24.1)	10 (21.7)
III	36 (25.4)	57 (39.3)	11 (23.9)
Pathology, n (%)			
Adenocarcinoma	125 (88.0)	133 (91.7)	45 (97.8)
SCC	17 (12.0)	11 (7.6)	1 (2.2)
Others	0 (0.0)	1 (0.7)	0 (0.0)
p53, n (%)			
0%	29 (20.4)	25 (17.2)	7 (15.2)
0%< to ≤10%	28 (19.7)	32 (22.1)	11 (23.9)
10%< to ≤50%	19 (13.4)	22 (15.2)	10 (21.7)
50%<	66 (46.5)	66 (45.5)	18 (39.1)
Adherence, n (%)	125 (88.0)	128 (88.3)	43 (93.5)

Continuous variables are presented as median (interquartile range) and categorical variables are

presented as number (percentage). Abbreviations: BMI, body mass index; SCC, squamous cell carcinoma; IHC, immunohistochemistry; pre25OHD, pre-study serum 25-hydroxyvitamin D level; 25OHD at 1 Year, serum 25-hydroxyvitamin D level at 1 year after study initiation

.

Table S3: Baseline Characteristics of Patients in the RR Genotype with p53 IHC Expression 50%< stratum

	Vitamin D	Placebo
N (%)	47 (71.2)	19 (28.8)
Age, years, median (IQR)	67 (61-75)	64 (59-72)
Sex, n (%)		
Male	34 (72.3)	14 (73.7)
Female	13 (27.7)	5 (26.3)
BMI, kg/m^2, median (IQR)	22.1 (20.3-24.0)	23.0(19.5-24.2)
Double cancer, n (%)	4 (8.5)	1 (5.3)
Pre25OHD, ng/mL, median (IQR)	22.0(16-27)	20.0(12-28)
25OHD at 1 Year, ng/mL, median (IQR)	48.5 (35-64)	20 (14-27)
Cancer sites, n (%)		
Esophagus	8 (17.0)	2 (10.5)
Stomach	11 (23.4)	8 (42.1)
Small bowel	0 (0.0)	0 (0.0)
Colorectal	28 (59.6)	9 (47.4)
Clinical stage, n (%)		
I	20 (42.6)	8 (42.1)
II	17 (36.2)	5 (26.3)
III	10 (21.3)	6 (31.6)
Pathology, n (%)		
Adenocarcinoma	39 (83.0)	17 (89.5)
SCC	8 (17.0)	2 (10.5)
Others	0 (0.0)	0 (0.0)
Adherance, n (%)	44 (93.6)	16 (84.2)

Continuous variables are presented as median (interquartile range) and categorical variables are presented as number (percentage). Abbreviations: BMI, body mass index; SCC, squamous cell carcinoma; pre25OHD, pre-study serum 25-hydroxyvitamin D level; 25OHD at 1 Year, serum 25-hydroxyvitamin D level at 1 year after study initiation

.

Table S4: Hazard ratios for relapse-free survival for vitamin D supplementation versus placebo in the subgroup of RR genotype with p53 IHC expression 50%< stratum, adjusted individually for clinical covariates

Covariate	HR	95% CI
Clinical stage	0.30	0.13-0.90
Pre25OHD	0.32	0.12-0.87
Cancer site	0.32	0.12-0.86

In the subgroup of the RR genotype with p53 IHC expression >50%, hazard ratios for relapse-free survival were evaluated by adjusting for individual clinical covariates. Abbreviations: HR, hazard ratio; CI, confidence interval; IHC, immunohistochemistry; Pre25OHD, baseline 25-hydroxyvitamin D levels.

Table S5: Hazard ratios for relapse-free survival for vitamin D supplementation versus placebo in the subgroup of RR genotype with p53 IHC expression 50%< stratum, stratified by cancer site

Subgroup	HR	95% CI
Esophagus	0.55	0.05-6.45
Stomach	0.23	0.05-0.98
Colorectal	0.48	0.08-2.89

Hazard ratios and 95% confidence intervals for relapse-free survival in the vitamin D group relative to the placebo group within the subgroup of RR genotype with p53 IHC expression 50%< stratum, stratified by primary cancer site (esophagus, stomach, and colorectal). Patients with small bowel cancer were not included in this subgroup. Abbreviations: RFS, relapse-free survival; HR, hazard ratio; CI, confidence interval; IHC, immunohistochemistry.

Table S6: Subgroup analysis of relapse-free survival for vitamin D supplementation versus placebo, stratified by *TP53* codon 72 genotypes (RR, PR, and PP) and p53 IHC expression stratum.

Subgroup		HR	95% CI
RR genotype	p53=0%	4.18	0.82-21.10
	0%<p53≤10%	0.61	0.04-9.93
	10%<p53≤50%	0.56	0.08-4.01
	50%<p53	0.32	0.12-0.87
PR genotype	p53=0%	0.84	0.22-3.13
	0%<p53≤10%	0.65	0.13-3.24
	10%<p53≤50%	0.43	0.05-3.85
	50%<p53	0.88	0.35-2.24
PP genotype	p53=0%	-	-
	0%<p53≤10%	-	-
	10%<p53≤50%	-	-
	50%<p53	0.66	0.15-2.96

Hazard ratios and 95% confidence intervals for relapse-free survival in the vitamin D group relative to the placebo group within each subgroup, categorized by *TP53* codon 72 genotypes (RR, PR, and PP) and p53 IHC expression status (0%, 0%<p53≤10%, 10%<p53≤50%, and 50%<p53). "-" indicates that hazard ratios could not be calculated due to zero events in one of the groups. Abbreviations: RFS, relapse-free survival; HR, hazard ratio; CI, confidence interval; IHC, immunohistochemistry.

Table S7: Somatic and Germline Genome of *TP53* Codon 72 Genotypes

		Germline (%)		
		RR	PR	PP
Somatic	RR	48(96.0)	6(10.2)	1(8.3)
	PR	0	44(74.6)	0
	PP	2(4.0)	9(15.3)	11(91.7)

Numbers represent the number of patients (percentage). Percentages are calculated within each germline genotype column.

Table S8: p53 IHC Staining and *TP53* Somatic Mutations

		p53 IHC (%)			
		0%	>0%< to ≤10%	>10% to ≤50%	50%<
Type of Mutation	Wild	13(59.1)	15(83.3)	13(65.0)	23(39.0)
	Missense	0	1(5.6)	5(25.0)	26(44.1)
	Nonsense	6(27.3)	2(11.1)	0	2(3.4)
	Deletion	2(9.1)	0	1(5.0)	4(6.8)
	Silent	1(4.6)	0	1(5.0)	4(6.8)

Numbers represent the number of patients (percentage). Percentages are calculated within each p53 IHC expression category column. Abbreviations: IHC, immunohistochemistry.

Figure S1: Sensitivity Analysis Subgroup of *TP53* codon 72 genotype and p53 IHC status

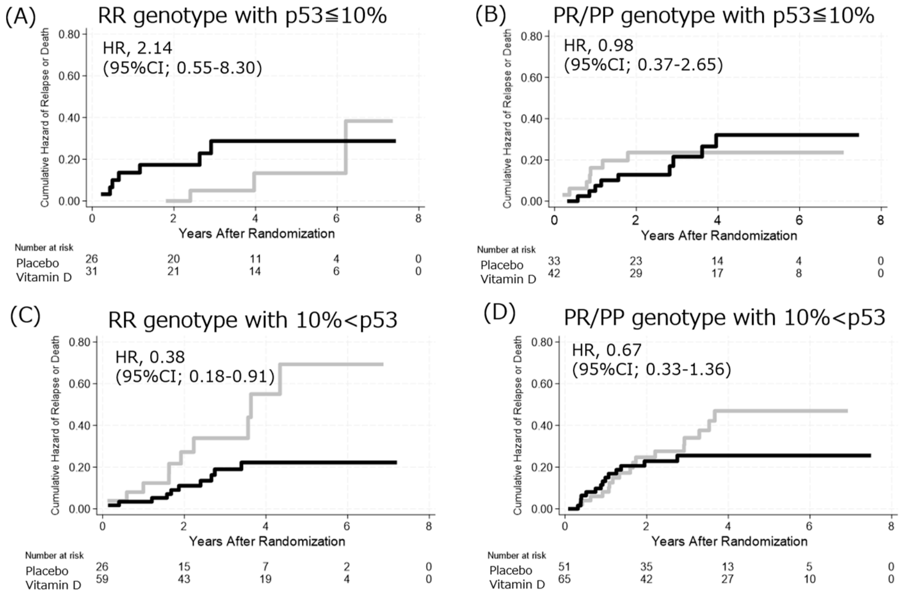

(A–D) Nelson–Aalen cumulative hazard curves for RFS in four patient subgroups defined by *TP53* codon 72 genotype and p53 protein IHC expression status: (A) the RR genotype with p53 ≤10%, (B) the PR/PP genotype with p53 ≤10%, (C) the RR genotype with 10%<p53, and (D) the PR/PP genotype with 10%<p53. In all panels, the black line represents the vitamin D supplementation group, and the gray line represents the placebo group. HRs and 95% CIs of the vitamin D group compared with the placebo group are indicated within each panel. RFS, relapse-free survival; HR, hazard ratio; CI, confidence interval.
